# Traditional Uses of Wild Edible Mushrooms among the Local Communities of Swat, Pakistan

**DOI:** 10.3390/foods12081705

**Published:** 2023-04-19

**Authors:** Shahid Hussain, Hassan Sher, Zahid Ullah, Mohamed Soliman Elshikh, Dunia A Al Farraj, Ahmad Ali, Arshad Mehmood Abbasi

**Affiliations:** 1 Center for Plant Sciences and Biodiversity, University of Swat, Swat 19120, Pakistan; 2 Department of Botany and Microbiology, College of Science, King Saud University, Riyadh 11451, Saudi Arabia; 3 Department of Environmental Sciences, Abbottabad Campus, COMSATS University Islamabad, Abbottabad 22060, Pakistan; 4 University of Gastronomic Sciences of Pollenzo, Piazza V. Emanuele II, 12042 Bra/Pollenzo, Italy

**Keywords:** ethnomycology, edible, medicinal mushrooms, ethnic community, Swat

## Abstract

Mushrooms play a crucial role in human life as well as in nature, providing food, medicine, and carrying out vital processes of decomposition, nutrient recycling, and developing mycorrhizal association with plants. The traditional system of knowledge about identification, collection, and usage of mushrooms has been accumulated through the shared experiences of many generations. Unfortunately, there have been continuous threats to the folk knowledge of mushrooms mainly due to habitat degradation, urbanization, and contemporary medication. The current research was, therefore, aimed to document an ethnomycological knowledge possessed by the ethnic communities of Swat, Pakistan. The purposive randomized sampling was carried out using chain referral method. Ethno-mycological information was collected from 62 informants using free listing, preference ranking, and use totaled methods. In total, 34 species of mushrooms belonging to 31 genera and 21 families were reported. About 85% of the reported species belong to Basidiomycetes, and 12.5% to Ascomycetes are used as food and for medicinal purposes. *Morchella angusticeps*, *M. esculenta*, *Pleurotus* sp., *Auricularia* sp., *Flammulina velutipes*, *Agaricus bisporus*, *Ganoderma lucidum*, and *Sanghuangporus sanghuang* were among the most cited edible and medicinal mushrooms. The current study revealed that district Swat is rich in wild edible and medicinal mushrooms (WEMs), and the local communities possess rich traditional knowledge about their collection, storage, and utilization. The diversity of WEMs of this region could contribute substantially to the socio-economic uplifting of the local communities through appropriate domestication and commercialization. Anthropogenic factors, coupled with depletion of traditional knowledge, threaten the diversity of WEMs in the region; therefore, in situ and ex situ conservation strategies are highly recommended.

## 1. Introduction

Mushrooms are an integral component of the forest ecosystem that carry out vital processes such as decomposition and nutrient recycling [1]. Most of the wild edible mushrooms (WEMs) serve as important supplementary or functional foods, whereas others have enormous medicinal potential [2,3]. Their use as food and part of the traditional medicine system dates back to ancient human civilization [4,5,6]. Some WEMs have been widely recognized as nutritionally important food with low caloric content, pleasant taste, and aroma. Studies reported that mushroom’s nutritional profile comprises high protein content, trace mineral elements, vitamins, fiber, and low fats contents providing good benefits to human wellbeing [7,8,9]. A considerable proportion of mushrooms have been used as food and are now scientifically proven to be healthier and safe for human consumption and are also considered to be an alternative to vegetables and many protein-rich foods [6,10]. Their biologically active ingredients are responsible for healing major human health conditions [11,12]. Collection of wild mushrooms is a very important activity for the livelihood in developing countries [4,13].

Approximately 14,000 mushrooms species have been identified, about 2000 of which are considered safe edible species [14]. Mushrooms are the principal component of biodiversity and constitute an economically important wild source of food and medicine for people around the world. This dependency established a deep and dynamic relationship between fungi and people across different cultural groups and thus became an inevitable part of the biocultural heritage [15]. The indigenous knowledge system and practices adopted through centuries are transferred from generation to generation through conversation and hence by large confronting threats of extinction or erosion unless used, passed, or documented properly [16]. The urbanization, introduction, and adoption of modern technology, loss of habitats due to alarming forest cutting, and linguistic and cultural dynamics are the factors that endanger biological and biocultural diversity [17,18]. The preservation of ethnomycological knowledge is important for drug prospects and validation through contemporary research techniques. The descriptive focus in such data has been completely changed to the recent quantitative ethnobiological approach [19]. According to Pérez-Moreno [20], it is crucial to consider the traditional knowledge of mushroom usage in modern studies to improve food security and prevent ignorance regarding their uses. Mushrooms, particularly morel species, serve as a source of food, medicine, as well as a valuable source of income for the local mountain dwellers in north Pakistan [21,22].

The dependency of tribal communities and ethnic societies on biological resources has been documented by several ethnobiological studies in the country [23,24,25,26,27,28,29,30,31,32,33]. Similarly, the economic impact of macrofungi and their cultural significance is evident in the studies of [34,35,36,37]. Additionally, also underprivileged inhabitants of the areas, particularly inaccessibly lying mountainous villagers, are still largely dependent on the traditional food and medical care system. Despite the rich diversity of medicinal and edible mushrooms in the country, knowledge about ethnomycological data has been rarely presented in the research. However, the scientific documentation of ethno-mycotaxa was unreported and critically ignored in the academic literature. In ethnomycological assessment, the use of quantitative approach was greatly neglected by many studies. The quantitative techniques in obtaining the ethnobiological data provided a reliable data set, robust analytical capabilities, and a higher level of confidence [38]. Only a few studies focused on traditional morphological, taxonomic, and ecological knowledge of mushrooms [39]. Hence, the present study aims to assess and inventory indigenous knowledge about mushroom uses, folk recipes, ecology, and folk taxonomy.

## 2. Materials and Methods

Swat is a district in northwest Pakistan, being a culturally and biologically diverse region [23]. Due to climatic and topographic variations, the area supports the growth of different macrofungi species of economic, medicinal, and cultural importance. The area has a mainly agroforest-based economy. The ecological zones ranging from subtropical to coldest alpine peaks with altitudinal range of about 600–4600 m. The indigenous communities that have narrow economic base are mainly dependent upon different agriculture and forest products [37]. Knowledge of the utilization of plants and mushrooms by the indigenous people is under reported.

For the current survey, rural villages inhabited by the major communities and producing mushrooms were selected. To validate the collected information and scrutinize the list of mushrooms for the onward survey, preliminary visits were undertaken to each locality during mushroom growth seasons i.e., March–May and then July–November. Due to the rare availability and collection of resources, the target group was randomly recruited for the survey. Sampling was carried out using referral sampling [40] and methods from [41,42]. Elite members from the tribe were asked to give a list of residential specialists and knowledgeable collectors; then, a random selection was made from the list. To document the folklore, beliefs, practices, and general knowledge about mushrooms species, semi-structured questionnaires were administered, and interviews were arranged with the resource collectors of different ages, educational levels, professional backgrounds, and experience levels. From each of the research participants, written prior informed consent (PIC) was obtained according to the Ethical Guidelines of the International Society of Ethnobiology (ISE). The research participants were the mushroom collectors and users mostly and consisted of 62 informants.

For interviews and questionnaires, designs from [43,44] were adopted. During the focus group discussion, we utilized the “totaled method” [45] to classify the various uses of mushrooms into five distinct categories: (1) Alimentary (edible), (2) Medicinal uses, (3) Economic (source of income), (4) Fungivory (mushroom seen to be consumed by insect, snails, maggots, rodents, or birds), and (5) Ecological (mushroom as rotter/decomposer of wood or litter). To quantify or rate the participants’ responses, a numeric scale was employed with “Yes” responses assigned a value of 1 and “No” responses as-signed a value of zero (0). Free listing technique was used for the collection of information regarding medicinal and food uses of mushrooms. To elicit information (12 key informants) regarding the ranking of 15 edible mushrooms, the preference scoring method was employed [41]. Five standard characteristics (smell, taste or flavor, distinctive texture such as softness, color, and allergic reaction) of food valuation were asked. Responses were used such as “good” 1, “bad” 0, “ordinary” 0.5, for allergic reaction “yes” 0, “No” 1. Based on the scale, the most preferable edible mushrooms were determined.

Questions were focused on the following aspects of the reported ethnotaxa, vernacular name, collection area, frequency of collection and self-consumption, localities of collection, medicinal and culinary usage, method of recipes preparation, physiologic effect, and remedial scale for alleviating symptoms. Furthermore, to corroborate the claims and authenticity of the information, the literature on ethnomycological data was consulted.

The specimens collected were identified using the standard mycological literature [46,47,48,49,50,51,52,53], and their taxonomic features were compared through morphoanatomical examination. Cross-comparison with previously submitted specimens in the mycological section of the herbarium at the University of Swat was used for further taxonomic validation. Websites such as Index Fungorum (http://www.indexfungorum.org, accessed on 5 February, 2023), Myco-Bank (http://www.mycobank.org, accessed on 5 February, 2023), and Mushroom Expert (https://www.mushroomexpert.com, accessed on 20 January, 2023) were accessed to confirm taxon nomenclature. For accurate species identification, maximum likelihood phylogenetic analyses were conducted using the aligned data set consisting of our generated rDNA ITS sequences along with retrieved reference sequences from NCBI GenBank (Supplementary Information S1). The identified specimens were dried and deposited at the University of Swat Herbarium (SWAT) under voucher numbers.

To investigate the underlying patterns in the responses of informants from four ethnic groups regarding the uses of mushrooms, a principal component analysis (PCA) using PC-ORD version 5 software was performed [54]. The data matrix included information on the diversity of mushroom uses reported by the informants. The PCA results were visualized using scatterplots, which identify clusters of mushroom uses that were commonly reported by different ethnic groups. Specifically, it was observed that the first two principal components accounted for a significant proportion of the variance in the data, and revealed distinct patterns of mushroom uses across different ethnic groups [55]).

Quantitative ethnomycological analyses were performed using different indices such as total use reports (TR), frequency of citation (FC), relative frequency of citation (RFC), and use value (UV). Total reports (TR) is the number of all uses attributed to a species as quoted by the informant’s information about folk use of a species [56]. Frequency of citation (FC) of mushroom species refers to the number of informants that mention the use of a particular mushroom species. This index is often used as a way to assess the cultural significance and versatility of a particular species. The relative frequency of citation (RFC) refers to the percentage of informants that mention the use of a particular species. It was calculated by using the formula of Tardio and Pardo-de-Santayana [57].

RFC = FC/N

where FC is the number of informants mentioning the use of a species and N is the total number of respondents.

The use value of plant species in ethnomedicine research is a measure of the relative importance or value of a plant species in the traditional medicinal practices of a specific cultural group or region. It is a quantitative method used to assess the significance of different plant species in a particular context [58].

UV = ∑ Ui/N

where UV represents use value, Ui is the number of uses mentioned by each informant for a given species, and N indicates total number of informants included in a survey.

## 3. Results

Results of the study were yielded by an extensive ethnomycological survey conducted during 2019–2022. Findings of the study are presented under the following headings.

### 3.1. Socio-Demographic Characteristics of Informants and Ethnic Groups

**Figure 1:**
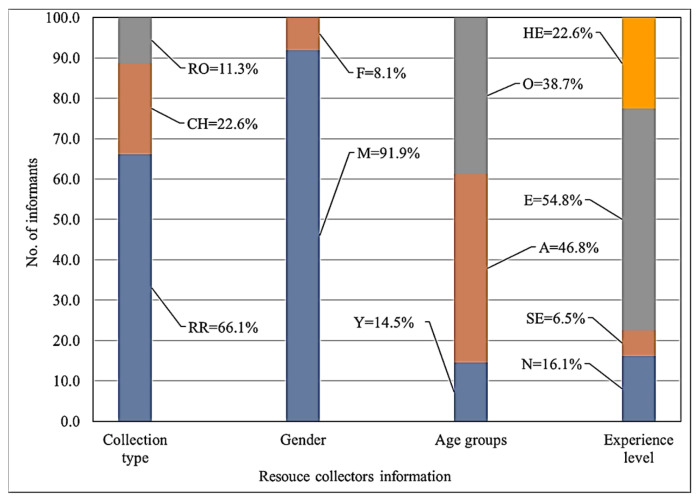
Resource collectors’ information. %: age of the resource collectors; RR: regular recreational harvesters; CH: commercial harvesters; RO: recreational and occasional harvesters; M: male; F: female; Y: youth; A: adult; O: elderly or old age; experience levels: N: novice, SE: somewhat experienced, E: experienced, HE: highly experienced.

WEMs traditional knowledge was obtained from 62 informants (= resource collectors) belonging to 17 villages and comprising 4 ethnic groups. Characteristics of the ethnic groups in the study area are shown in Table 1. The informants included 91.9% male participants and 8.1% female participants. The informants belonged to different age groups as shown in Figure 1. More than 90% of the informants belonged to the age class above 20 years showing a good level of field experience. The informants mainly consisted of males. Because of religious taboo to participate in the survey and infrequent involvement in mushroom collection due to parenting and household responsibilities, only five of the old age women participated in the study.

The resource collectors (Figure 1) had different livelihood sources which were generally related with the existing agroforest-based economic system of the area. The resource collectors comprised individuals of different age groups; however, the dominant age was adults between 26 and 60 followed by elderly age (>60) individuals. However, young children were frequently observed to collect wild food and edible mushroom species, MAPs, and other forest products. The resource collectors were classified into commercial harvester, recreational regular, and occasional collector. Regarding their experience level, the highly experienced individuals were predominant in the current study.

**Figure 2:**
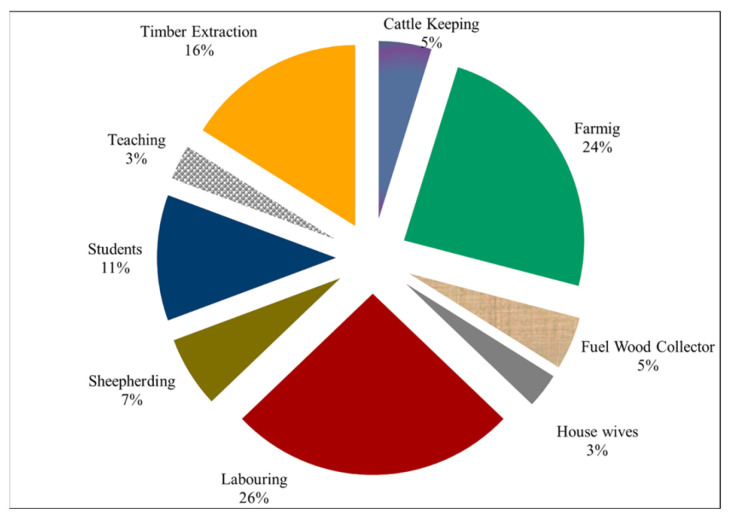
Distribution of the informant’s occupation categories.

The occupations of local informants consisted of farming, cattle raising, horticulturing, sheepherding, and forest resource collection (Figure 2). The number of ethnotaxa reported by each informant range from 2 to 21 spp. (average = 10.17), whereas total mentioned reports range from 4 to 46 by an individual informant. The correlation coefficient between the age of collector and number of cited taxa was calculated to be weak positive (0.0895) and non-significant.

**Figure 3:**
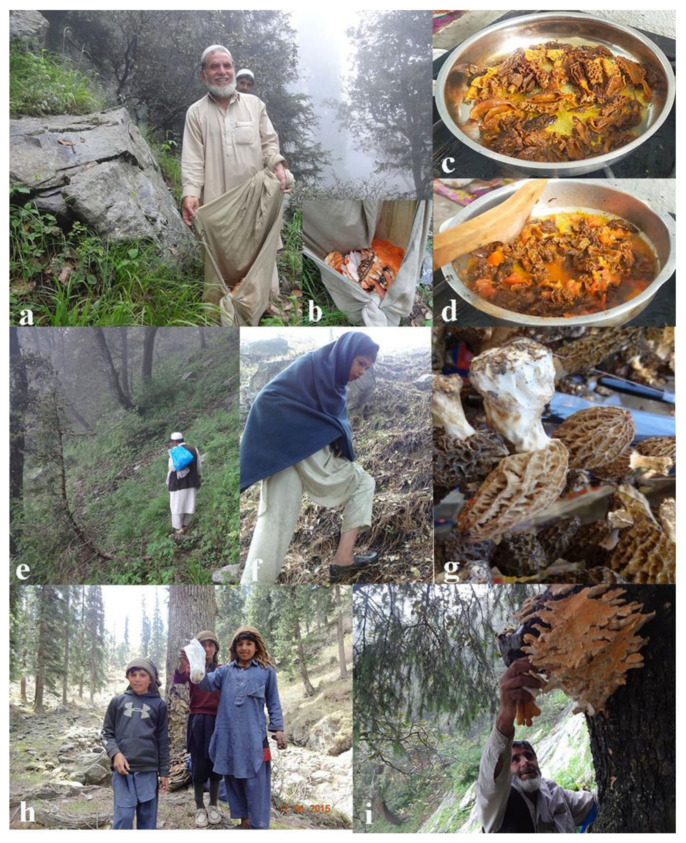
Resource collectors from the study area (**a**,**e**,**f**,**h**,**i**), (**b**) *L. zonatus* transporting in cloth sheet, (**c**) food preparation morels frying in oil, (**d**) food preparation of morels mixed with tomato, (**g**) morel fruiting bodies using hang with threads, (**i**) collector hand picking *L. zonatus* fruiting bodies. The participants were well aware of the purpose of the study, and were informed about how the images would be used/produced. They were also informed that the photos may be used for the purpose to share the knowledge of mushroom among the global community, and that they had the right to revoke their consent at any time.

The resource harvesters were involved in the collection, cleaning, utilization, preservation, and marketing of their stock collection (Figure 3). The majority of the research participants (80.5%) collect mushrooms in local forests, farms, and plantations surrounding the villages, whereas 19.5% of the collectors also visit forests located in other regions.

### 3.2. Biological Spectrum of the Ethnotaxa

The survey revealed that people in the study area were using a total of 34 mushrooms species belonging to 21 families, which further consist of Basidiomycetes (29) and Ascomycetes (5). Among all families, the dominant families were Hymenochaetaceae (14.7%) and Polyporaceae (11.8%), followed by Lycoperdaceae (8.2%), Morchellaceae (8.2%), Physalacriaceae (5.9%), and Agaricaceae (5.9%); other families were represented by single mushroom species each.

### 3.3. Ethnicity and Cultural Profile

Mushroom indigenous knowledge was obtained from four different ethnic groups. These communities were classified into Pakhtun community (PC), Pakhtun-Gujjar community (PGC), Pakhtun-Kohistani community (PKC), and Kohistani community (KC). Striking differences regarding folk information, traditional belief, and practices about ethnotaxa were observed among all groups. On an average basis, the number of mushroom species cited by an individual participant from each community to have some ethnomycological importance were calculated as PGC (14 spp.), PKC (10 spp.), PC (8 spp.), and KC (7 spp). The maximum number of species and citations were recorded from PGC (33 spp., 490 citations), followed by PKC (28 spp., 212 citations), KC (26 spp., 221 citations), and PC (20 spp., 126 citations).

**Figure 4:**
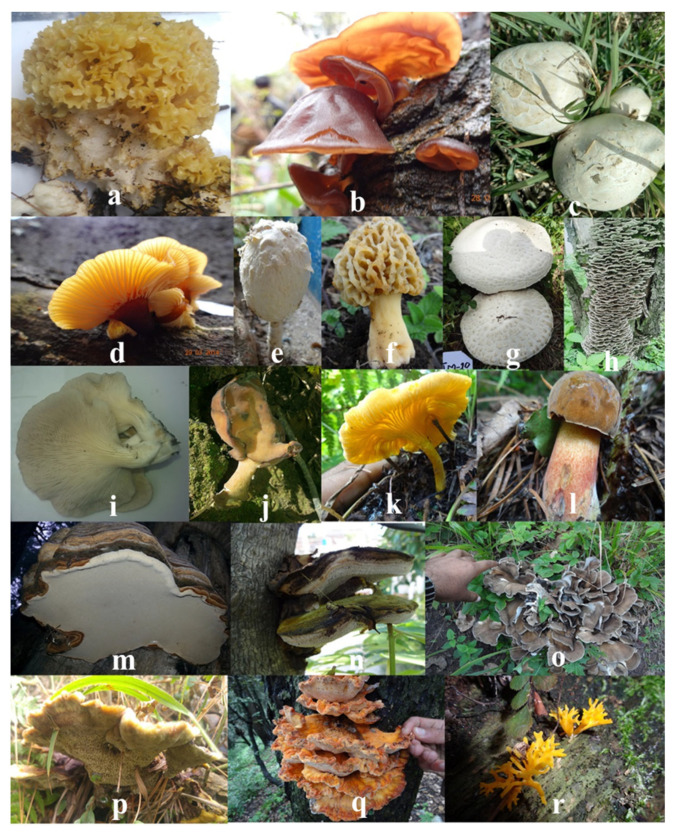
Mushroom fruiting bodies used as ethnomedicine and edible mushrooms: (**a**) *S. latifolia*, (**b**) *Auricularia* sp., (**c**) *Calvatia* sp., (**d**) *F. velutipes*, (**e**) *C. comatus* (**f**) *M. esculenta*, (**g**) *A. bisporus* (**h**) *T. versicolor* (**i**) *Pleurotus* sp. (**j**) *Helvella* sp., (**k**) *C. cibarius* (**l**) *B. edulis* (**m**) *F. fomentarius*, (**n**) *Innonotus* sp. (**o**) *G. frondosa*, (**p**) *P. schweinitzii*, (**q**) *L. zonatus* (**r**) *C. viscosa*.

Some highly utilized species of mushrooms used by local communities as food and medicine are illustrated in Figure 4. Based on the number of higher reports regarding edibility, the most widely used species from PC community were *M. angusticeps*, *M. esculenta*, *Rhizopogon* sp., *Auricularia* sp., *F. velutipes*, *A. bisporus*, and *P. squamosus*, whereas from PGC, the most widely consumed species were *M. angusticeps*, *M. esculenta*, *M. angusticeps*, *L. zonatus*, *C. cibarius*, *Auricularia* spp., *H. cirrhatum*, and *Pleurotus* sp. Similarly, from PKC, the prime edible species were found to be *M. angusticeps*, *M. esculenta*, *Pleurotus* sp., *Auricularia* sp., *F. velutipes*, and *S. latifolia*, *A. bisporus*, and from the KC community, *M. angusticeps*, *M. esculenta*, *Calvatia* sp., *Ramaria* spp., *S. latifolia*, and *A. bisporus* were the commonly used edible mushrooms.

### 3.4. Folk Mushroom Knowledge

The increasing awareness of the significant role that mushrooms play in medicine and myco-gastronomy has led to the extensive documentation of numerous useful mushroom species from different parts of the world. In the present work, the local harvesters classified mushrooms into four categories: edible, inedible, medicinal, and poisonous mushrooms. Boa [2] has reported a somewhat similar categorization. The traditional nomenclature and identification system used by harvesters to communicate about these mushrooms relies mainly on organoleptic evaluation and visual inspection of the fruiting bodies to distinguish poisonous from non-poisonous mushrooms. The local perception system helps in confirming the identification of mushrooms. Health risks or fatalities due to misidentification, carelessness in handling, or consumption of mushrooms are very unlikely, as the indigenous communities have developed effective identification techniques. However, myths, personal opinions, or community general perceptions about the utilization of mushrooms have been reported in several studies [54,59]. These perceptions require scientific validation and assessment to distinguish fact from fiction.

*Poisonous mushrooms:* These mushrooms are known as dog’s food or rabies mushrooms and are referred to by local inhabitants as “Spoo Kharary”. They can be identified by:

- Red-, pink-, purple- or yellow-colored caps (30 reports) and swollen stalk base (2 reports);
- They have a bad smell, bitter taste (5 reports), and causes numbness to the tongue and lips (13 reports);
- They repels/deter insect, worms, and rodents (9 reports);
- They secrete milky fluids (1 report).

The literature shows some of these characters are exhibited by the poisonous mushrooms belonging to *Amanita* spp. *Cortinarius* spp. *Galerina marginata*, some *Conocybe* spp., *Gyrometra* spp., and some of *Lactarius* and *Entoloma* spp. According to harvesters, there are about 1–2 cases each year of severe intoxication or toxin syndrome due to ingestion of poisonous mushrooms, mostly among novice hunters or children.

*Edible mushrooms:* Mushrooms that are safe and suitable to consume without causing any health risks are known as locally “Khorak Kharary”, and they can be identified by:

- They are typically fleshy and soft (39 reports);
- White caps (60 reports);
- They are known to attract flies, worms, and snails (10 reports);
- Reported to have pleasant taste like fish or chicken (52 reports);
- These mushrooms usually grow on *Ficus* spp. (51 reports), *Morus* spp. (22 reports), *Ailanthus altisimma* (21 reports), and *Populus* spp. (4 reports).

The local inhabitants/collectors rely on traditional knowledge to minimize the risk of factors of mushroom poisoning or mild intoxication, and to neutralize mushroom toxicity. Various methods are used for this purpose, including:

- Overnight soaking in hot water followed by refrigeration (3 reports);
- Boiling and overcooking of mildly poisonous mushrooms mostly *Helvella* spp., *Gyromitra* spp. (12 reports);
- Salt roasting and mixing with vegetables (2 reports);
- Use of milk or vinegar to reduce the toxic effect (3 reports).

Rubel and Arora [60] have demonstrated that the potential toxin in *Amanita muscaria* can be reduced through pre-treatment and cooking. Overall, the local inhabitants employ these techniques to ensure that the mushrooms they consume are safe and free from any harmful effects.

### 3.5. Traditional Uses of Mushrooms

Traditional uses of reported species of mushrooms are given in Table 2. A total of 26 species were used for culinary purposes, whereas 8 species were used in traditional medicine system (Table 2). Among the edible mushrooms, five of the species were shown to have both nutritional and medicinal properties. Using the acquired knowledge, mushroom foraging was carried out during two seasons of the year, March to May and July to November. Targeted zones or patches with ecoclimatic signatures were used by the collectors to locate mushrooms. Some of the perennial fruiting bodies were available throughout the year but their collection was preferred during spring and rainy season of the year. Two species viz. *F. velutipes*, *Rhizopogon* sp. were usually collected during late winter and early spring seasons. Among the edible mushrooms, 53.8% (14) were collected in their young or non-sporulating stage, while 3 species such as *H. cirrhatum*, *B. edulis*, and *P. squamosus* were harvested only in young stage because their mature fructifications were infested and decimated by frugivorous pest.

Recipes of culinary and therapeutic mushrooms are given in Table 2. Eight of the edible mushrooms were collected only in their mature stage. For the sake of max profit, commercial species such as *Morchella* spp. were mostly collected both in young and mature stages. By contrast, all medicinal species were harvested in their mature condition. A total of 73.5% of the species were used in fresh condition, while 11.8% were used dried and the other 14.7% were used both in fresh and dried condition.

The higher FC value was recorded for *M. angusticeps* (95.2%), *M. esculenta* (91.9%), and *Pleurotus* sp. (51.6%). Other species, where the FC <40, consisted of *Auricularia* sp. (48.4%), *F. velutipes* (45.2%), *A. bisporus* (43.5%), *C. comatus* (40.3%), and *C. cibarius* (40.3%) (Table 3). Among the edible mushrooms total number of citations in all ethnomycological categories, the maximum citations were received by *M. esculenta* (116), *M. angusticeps* (113), *F. velutipes* (54), *H. cirrhatum* (46), *Auricularia* sp. (43), *A. bisporus* (41), *G. lucidum* (40), and *Calvatia* sp. (40). For medicinal species, higher citations were shown by *M. angusticeps* (16), *S. sanghuang* (100), *Fuscoporia* sp. (9), and *G. lucidum* (7). For five food characteristic categories, rating was performed by the 12 key informants, the highest scores among which were gained by the *Morchella* spp. (41), *S. latifolia.* (38.5), *Pleurotus* sp. (36.5), *L. zonatus* (32.5), and *C. cibarius* (32) (Table 4).

**Figure 5:**
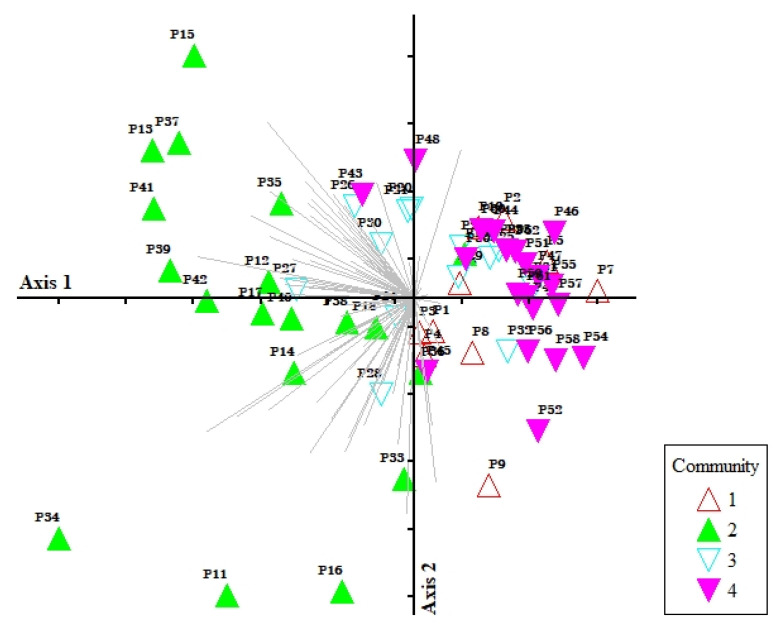
PCA graph illustrating the variation of information among ethnic communities in relation to 34 wild mushrooms. Axis 1 (component 1) and Axis 2 (component 2) represent the principal components. The legend indicates the ethnic communities, with ‘P’ representing the participants of the study. 1 refers to the Pakhtun community, 2 represents the Pakhtun-Gujjar community, 3 denotes the Pakhtun-Kohistani community, and 4 represents the Kohistani community.

The responses provided by the informants from four ethnic groups were depicted on a principal component graph (Figure 5). The analysis indicated that axis 1 received variation of 11.3% while axis 2 had 6.8%. The data showed that PK, P, and K responses were more or less similar, whereas the PG responses had great variations.

## 4. Discussion

The present study collected data from four ethnic communities residing within mountainous areas of the district Swat. Indigenous communities in the area consider mushrooms an important source of supplementary food and medicine. Furthermore, data from recent studies by Ullah et al. [61] and Hussain and Sher [37] have indicated the vast amount of mushroom collection and consumption among the local people. In the current survey, a total of 26 species were used for culinary purposes, whereas 8 species were used in traditional medicine system. Similar to other countries such as Nigeria, Nepal, Mexico, and India, mushrooms are an alternative protein source for indigenous and underprivileged communities in our study area [62,63,64,65]. Despite the presence of a diverse range of valuable fungal species in the region, their potential uses remain largely unreported and undocumented, hindering our understanding of their true value.

The utilization and number of ethnotaxa used as food and in traditional healthcare systems vary among all four communities, indicating the presence of cultural dynamics and variation. As a result, different mushroom species are treated differently [19]. This system of knowledge has developed due to the constant interaction between people and fungi in response to the climatic and environmental features of the area [66]. Moreover, our study reveals that indigenous mushroom knowledge varies based on the socio-economic factors and demographic attributes of the population, such as age, sex, and economic status [67]. The findings highlight the significance of taking into account the cultural and socio-economic factors when studying the traditional use of mushrooms.

The distribution and practice of local knowledge are influenced by various variables, including gender. Our study on ethnomycological knowledge has revealed the significant role of women in this field [68,69]. They actively participate in mushroom collection, utilization, and aid the seasonal household economies by using mushroom as supplementary dietary source. Furthermore, women’s predominant roles in household management, such as cooking, childcare, and disease management, have enabled them to hold a substantial amount of ethnobiological knowledge [23]. However, the gender imbalance in our study is due to religious and patriarchal family structures, which prioritize males as family heads and resource holders [70]. Consequently, we only included a limited number of female participants due to societal norms and religious taboos.

Our study revealed that elderly members of the community who possess knowledge of mushroom identification, ethnomedicinal, and ecological knowledge of mushrooms are more reliable. More than 90% of the informants were above 20 years old, indicating a good level of experience spanning multiple generations. Similar ethnomycological knowledge was also reported in previous studies [64,65,70]. The practice of locating and collecting useful mushrooms in the forests requires an adequate understanding of the spatial and temporal distribution and ecological knowledge possessed by the resource collector. Similar trends were also observed in other studies [70,71]. In the present study, the knowledge of folk taxonomy and nomenclature gained from elderly people in the community is considerably similar to [15,72]. The identification of mushrooms was performed using organoleptic evaluation and traditional techniques, which were also used in other countries [72]. Mostly, color, shape, smell, host plant, or substrate were the criteria used for identifying the species. Other studies have also reported similar local identification techniques [39,63,64,65]. Our study found that the quantum and frequency of collection depended on factors such as growth habit, gregarious or caespitos behavior, fructification, and substrate availability in the collection area. Overcooking, mixing with vegetables, and heat and salt neutralization, as well as fresh use of perishable mushroom food, were also recorded in other studies, such as [39,63,72].

In this study, mushrooms were recorded and evaluated for their therapeutic relevance and their use in the treatment of some of the common ailments including diabetes, cancer, hypertension, hyper level of cholesterol, etc. Despite their promising therapeutic applications, the use of mushrooms in ethnomedicine remains relatively uncommon compared to other fields. This may be due in part to concerns about mushroom poisoning incidents, which occur at a rate of approximately one to two cases per year. Additionally, further pharmacological validation and experimentation are necessary to fully explore the potential of mushrooms in ethnomedical practices [73].

## 5. Conclusions

Mushrooms are essential to both humans and the environment, serving as a source of food, medicine, and nutrients, as well as facilitating crucial processes such as decomposition and nutrient recycling. Indigenous communities possess a rich knowledge of wild mushroom collection, handling, storage, and utilization, with this knowledge varying significantly among different groups. In developing countries, wild edible mushrooms (WEMs) are a valuable alternative to protein foods, making them essential in combating food insecurity. For entrepreneurs in the food and medicine industries, ethnobiological knowledge can aid in identifying new food sources and products, as well as validating traditional medicine practices through scientific experimentation. Therefore, further exploration of mushroom knowledge using contemporary scientific tools is necessary for socio-economic growth. It is crucial to recognize and appreciate traditional knowledge for socio-economic development. However, mycological resources and their significance in forest ecosystems have been largely overlooked in management and conservation policies. To devise more locally relevant conservation priorities, measuring the cultural importance of ethno-taxa can be useful. Unfortunately, the vertical transmission of ethnobiological knowledge from generation to generation is hampered by modern technologies, and younger generations may not be interested in acquiring or transmitting this knowledge. Therefore, alternative methods of knowledge transfer should be explored to ensure the preservation and utilization of traditional knowledge for future generations.

## Figures and Tables

**Table 1:** Ethnic groups, their geographical distribution, and associated forest types.

Communities	Code	Villages	FT	TI	MR
Pakhtun	P	Kokarai, Jambil, Bandai, Marghazar, Banjot	Subtropical deciduous forests	10	20
Pakhtun-Gujjar	PG	Malam Jabba, Miandam, Lalku, Sailand	Mixed conifers moist temperate	19	33
Pakhtun-Kohistani	PK	Chail, Bashigram, Pian, Shinko	Mixed conifers moist temperate	13	28
Kohistani	K	Behrain, Kalam, Ushu, Gabral	Cedar conifers dry temperate	20	26

TI: total informants; MR: number of mushrooms reported; FT: forest type.

**Table 2:** Traditional food and medicinal uses of WEMs, local names, seasonal availability, and voucher information.

Scientific Name/Local Name/Family	Uses	Season	Medicinal	Recipe
*Trametes versicolor* (L.) Lloyd Patai Kharary Polyporaceae SWAT002001	Med, Mt, D, Gg	July–December	Respiratory	Drying and smoke inhalation
*Sanghuangporus sanghuang Sheng* H. Wu, L.W. Zhou & Y.C. Dai Largi Kharary Hymenochaetaceae SWAT002002	Med, Mt, D, Sg	Perennial	Immunomodulatory, antipyretic, anticancerous	Dried, ground used as powder, tea
*Polyporus squamosus* (Huds.) Fr. Spardar Kharary Polyporaceae SWAT002003	Ed, Yg, F, Sg	August–November		Fried in oil
*Fomes fomentarius* (L.) Fr. Largi Kharary Polyporaceae SWAT002004	Med, Mt, F, Sg	Perennial	Antidiabetic, antiarthritic, anticancerous, used as poultice	Powdered and boiled in water
*Fuscoporia* sp. Soor Kharary Hymenochaetaceae SWAT002005	Med, Mt, D, Ca	Perennial	Fatigue and antipyretic, general tonic	Powdered, mixed with honey
*Laetiporus zonatus* B.K. Cui & J. Song Dodai Laetiporaceae SWAT002006	Ed, Yg, F, Ca	August–September		Spiced and fried in oil, boiled and mixed with vegetables
*Grifola frondosa* (Dicks.) Gray Charg lakai Grifolaceae SWAT002007	Ed, Yg, F, Gg	August–September		Fried in oil, boiled with meat to prepare soup
*Phellinus nigricans* (Fr.) P. Karst. Largi Kharary Hymenochaetaceae SWAT002008	Med, Mt, D, Sg	Perennial	Anticancerous, immunomodulatory, antihelminthic, antispasmodic	Powdered and boiled in water to prepare decoction, as poultice
*Sparassis latifolia* Y.C. Dai & Zheng Wang Gopi panra Sparassidaceae SWAT002009	Ed-Med, Yg, F, Gg	July–August	General body tonic	Soup preparation, fried in oil, mixed with vegetable, boiled to use in salad
*Volvariella bombycina* (Schaeff.) Singer Yakhta Kahrary Pluteaceae SWAT002010	Ed, Mt, F, Sg	July–November		Oil frying
*Auricularia* sp. Gwag Kharary, Gal Auriculariaceae SWAT002011	Ed-Med, Mt, F, Sg	March–August	Laxative, astringent, common cold, antihypertensive	Cooked thoroughly in oil, mixed with honey and walnut
*Flammulina velutipes* (Curtis) Singer Jami Kharary, Inzar Kharary Physalacriaceae SWAT002012	Ed, Mt, F, Ca	January–April		Cooked in oil, soup preparation mixed with vegetables and chicken meat
*Gyromitra* sp. Wrana Gujai Discinaceae SWAT002013	Ed, Mt-Yg, F, Sg	March–September		Boiled to remove color then applied with spice and fried in oil
*Morganella pyriformis* (Schaeff.) Kreisel & D. Krüger Ball Lycoperdaceae SWAT002014	Ed, Yg, F, Gg	July–November		Fried in oil
*Morchella esculenta* (L.) Pers. Ziara Gujai, Cashind Ghesi Morchellaceae SWAT002015	Ed-Med, Yg-Mt, F-F, Gg	March–May	Tonic, aphrodisiac	Fried in oil, soup and sauce, hot food
*Morchella angusticeps* Peck Tora Gujai Morchellaceae SWAT002016	Ed-Med, Yg-Mt, F-F, Gg	March–May	Tonic, immune stimulatory function	Fried in oil, soup and sauce, hot food
*Agaricus bisporus* (J.E. Lange) Imbach Pasheer ghasi Agaricaceae SWAT002017	Ed, Yg, F, Gg	March–September		Fried in oil, mixed with butter
*Lycoperdon perlatum* Pers. Da bodai Naswar, Dogh Lycoperdaceae SWAT002018	Ed, Yg, F, Gg	July–November		Fried in oil, mixed with potato
*Calocera viscosa* (Pers.) Fr. Ziar Khkary Dacrymycetaceae SWAT002019	Md, Mt, F, Sg	September–November	Eyesight, laxative	Wetted in water and rubbed on eyeballs
*Hericium cirrhatum* (Pers.) Nikol. Shalgotai Hericiaceae SWAT002020	Ed, Yg, F, Sg	July–September		Cooked with oil and mixed with cheese
*Verpa conica* (O.F. Müll.) Sw. Topai, Kochor top Morchellaceae SWAT002021	Ed, Yg, F, Sg	March–May		Fried in oil
*Helvella* sp. Khar Ghwag, Gal Helvellaceae SWAT002022	Ed, Yg, F, Sg	March–September		Fried in oil or mixed with vegetables
*Armillaria* sp. Gal Physalacriaceae SWAT002023	Ed, Mt, F, Gg	July–September		Fried in oil
*Cantharellus cibarius* Fr. Ziar Kharary Hydnaceae SWAT002024	Ed, Mt, F, Sg	August–September		Fried in oil or mixed and boiled with vegetables
*Lactarius deliciosus* (L.) Gray Zmaky Kharary Russulaceae SWAT002025	Ed, Yg, F, Sg	July–September		Fried in oil
*Pleurotus* sp. Inzar Kharary Pleurotaceae SWAT002026	Ed, Mt, F, Ca	July–September		Fried in oil, soup and sauce preparation, mixed with vegetables
*Boletus edulis* Bull. Sponge Boletaceae SWAT002027	Ed, Yg, F, Sg	July–November		Flesh from the young cap fried along with potato chips
*Coprinus comatus* (O.F. Müll.) Pers. Speen Kharary Agaricaceae SWAT002028	Ed, Yg, F, Sg	March–September		Fried in oil or daisy ghee
*Phaeolus schweinitzii* (Fr.) Pat. Largi Kharary Hymenochaetaceae SWAT002029	Med, Mt, F/D, Sg	July–November	Anthelminthic, or used as disinfectant, used in skin rashes	A drop or two squeezed in cup of water
*Inonotus* sp. Ziar Kharary Hymenochaetaceae SWAT002030	Med, M, F/D, Sg	March–September	Antidiabetic, anticancerous	Powdered and boiled to prepare tea
*Ganoderma lucidum* (Curtis) P. Karst. Makhoka kharary, Soor kaharary Polyporaceae SWAT002031	Ed-Med, Yg/Mt, F/D, Gg	July–November	Immune stimulatory, antipyretic, anticancerous	Tea, young fruiting are spiced and fried
*Calvatia* sp. Bodai ball, Mola Dongur Lycoperdaceae SWAT002032	Ed, Yg, F, Gg	August–November		Fried in oil
*Ramaria* sp. Gotai Kharary Gomphaceae SWAT002033	Ed, Yg, F, Ca	August–November		Fried in oil
*Rhizopogon* sp Kachaloo Kharary Rhizopogonaceae SWAT002034	Ed, Yg, F, Gg	January–March		Fried in oil

Med: medicinal; Ed: edible; Yg: used as young FB; Mt: mature FB; F: used fresh, D: used dried; Gg: gregarious; Ca: caespitose; Sg: singly.

**Table 3:** Relative frequency of citation, use categories, use reports, and use value of reported mushrooms.

S. No	Ethnotaxa	TR	FC	RFC	No. of Citation per Category					UR	UV
					Alimentary	Medicinal	Economic	Fungivory	Ecological		
1	*T. versicolor*	1	1.6	0.0	0	1	0	0	0	1	0.02
2	*S. sanghuang*	10	16.1	0.3	0	10	0	0	3	13	0.21
3	*P. squamosus*	21	33.9	0.5	21	0	0	6	12	39	0.63
4	*F. fomentarius*	6	9.7	0.2	0	6	0	1	4	11	0.18
5	*Fuscoporia* sp.	9	14.5	0.2	0	9	0	0	2	11	0.18
6	*L. sulphureus*	17	27.4	0.4	17	0	1	8	5	31	0.50
7	*G. frondosa*	16	25.8	0.4	16	0	0	0	6	22	0.35
8	*P. nigricans*	3	4.8	0.1	0	3	0	0	2	5	0.08
9	*S. latifolia*	21	33.9	0.5	21	5	0	1	11	38	0.61
10	*V. bombycina*	19	30.6	0.5	19	0	0	2	8	29	0.47
11	*Auricularia* sp.	30	48.4	0.8	30	2	0	0	9	41	0.66
12	*F. velutipes*	28	45.2	0.7	28	0	2	3	13	46	0.74
13	*Gyromitra* sp.	15	24.2	0.4	15	0	0	0	7	22	0.35
14	*M. pyriformis*	14	22.6	0.4	14	0	0	0	6	20	0.32
15	*M. esculenta*	57	91.9	1.5	57	2	32	10	15	116	1.87
16	*M. angusticeps*	59	95.2	1.5	59	16	12	17	9	113	1.82
17	*A. bisporus*	27	43.5	0.7	27	0	6	0	7	40	0.65
18	*L. perlatum*	21	33.9	0.5	21	0	0	0	10	31	0.50
19	*C. viscosa*	2	3.2	0.1	0	2	0	0	1	3	0.05
20	*H. cirrhatum*	19	30.6	0.5	19	0	0	11	13	43	0.69
21	*V. conica*	10	16.1	0.3	10	0	0	0	6	16	0.26
22	*Helvella* sp.	15	24.2	0.4	15	0	0	2	8	25	0.40
23	*Armillaria* sp.	13	21.0	0.3	13	0	0	0	4	17	0.27
24	*C. cibarius*	25	40.3	0.7	25	0	4	0	0	29	0.47
25	*L. deliciosus*	15	24.2	0.4	15	0	0	7	4	26	0.42
26	*Pleurotus* sp.	32	51.6	0.8	32	0	4	0	18	54	0.87
27	*B. edulis*	16	25.8	0.4	16	0	0	10	6	32	0.52
28	*C. comatus*	25	40.3	0.7	25	0	0	0	8	33	0.53
29	*P. schweinitzii*	2	3.2	0.1	0	2	0	2	2	6	0.10
30	*Inonotus* sp.	5	8.1	0.1	0	5	0	0	5	10	0.16
31	*G. lucidum*	22	35.5	0.6	22	7	0	2	9	40	0.65
32	*Calvatia* sp.	22	35.5	0.6	22	0	5	1	11	39	0.63
33	*Ramaria* spp.	24	38.7	0.6	24	0	0	0	12	36	0.58
34	*Rhizopogon* sp.	11	17.7	0.3	11	0	0	0	0	11	0.18

TR: total reports; FC: frequency of citation; RFC: relative frequency of citation; UR: use report; UV: used value.

**Table 4:** Scoring of edible mushrooms performed by the 12 key informants.

SN	Species	Smell	Taste or Flavor	Distinctive Texture	Color	Any Discomfort or Allergic Reaction	Total Score Gained
1	*Morchella* spp.	7	9	9	7	9	41
2	*S. latifola*	7.5	8	7	9	7	38.5
3	*Pleurotus* sp.	5	8.5	7	8	8	36.5
4	*L. zonatus*	5.5	8	6	6	8	33.5
5	*C. cibarius*	4	7	8	7	7	33
6	*P. squamosus*	5	7.5	7.5	7	6	33
7	*A. bisporus*	5	8	6	6	7	32
8	*F. velutipes*	3.5	8	6.5	7	4.5	29.5
9	*H. cirrhatum*	6	7.5	7	5	4	29.5
10	*Auricularia* sp.	5	4	7	7	5	28
11	*G. frondosa*	6	6.5	3	5	6.5	27
12	*Ramaria* spp.	4	5.5	4	6	7	26.5
13	*Calvatia* sp.	3.5	4	4.5	4	7	23
14	*L. perlatum*	4	4	5	4	4	21
15	*V. bombycina*	2	4	4	4	6	20

## Data Availability

All data are available in this manuscript; however, for additional information, corresponding authors may be consulted.

## Supplementary Materials

The following supporting information can be downloaded at: https://www.mdpi.com/article/10.3390/foods12081705/s1, Supplementary Information S1: Phylogenetic trees and morphological description of species.
