# Supplementary material for: Traditional Uses of Wild Edible Mushrooms among the Local Communities of Swat, Pakistan
Source: Foods. 2023 Apr 19;12(8):1705. doi: 10.3390/foods12081705 (PMC10137476; doi:10.3390/foods12081705)
Supplement: Supplementary file 1 [file foods-12-01705-s001.zip › foods-2249625-supplementary.pdf]

S1: Phylogenetic trees and morphological description of mushroom species

1. *Trametes versicolor* (L.) Lloyd

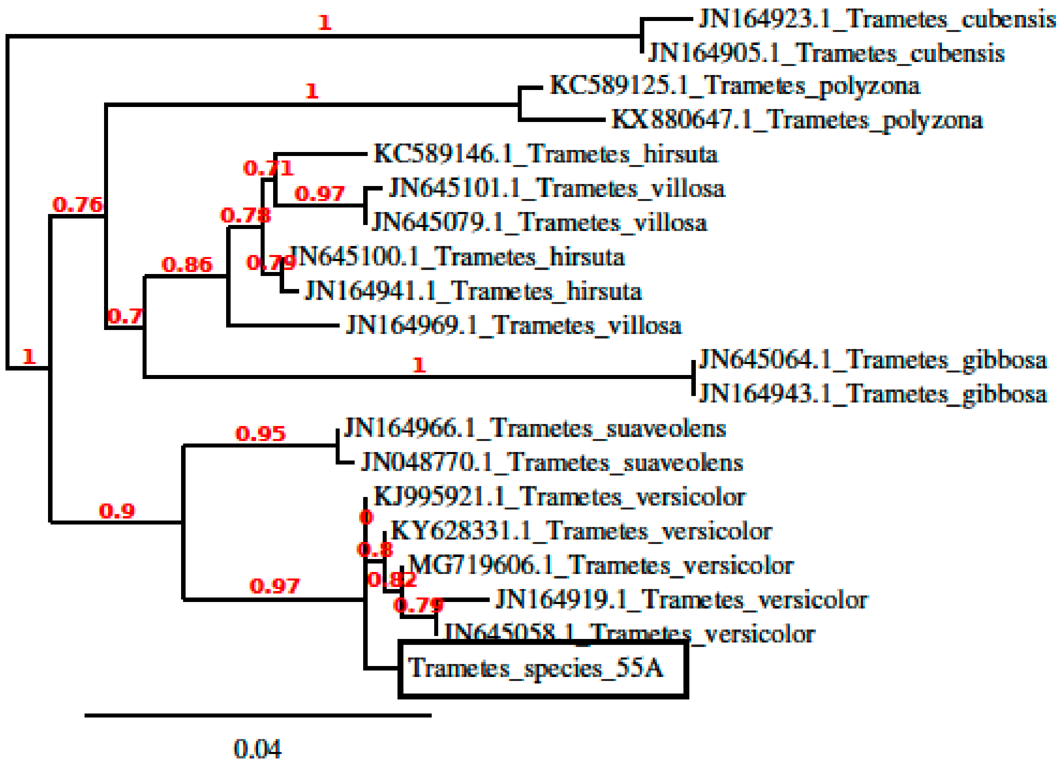

2. Sanghuangporus sanghuang Sheng H. Wu, L.W. Zhou & Y.C. Dai

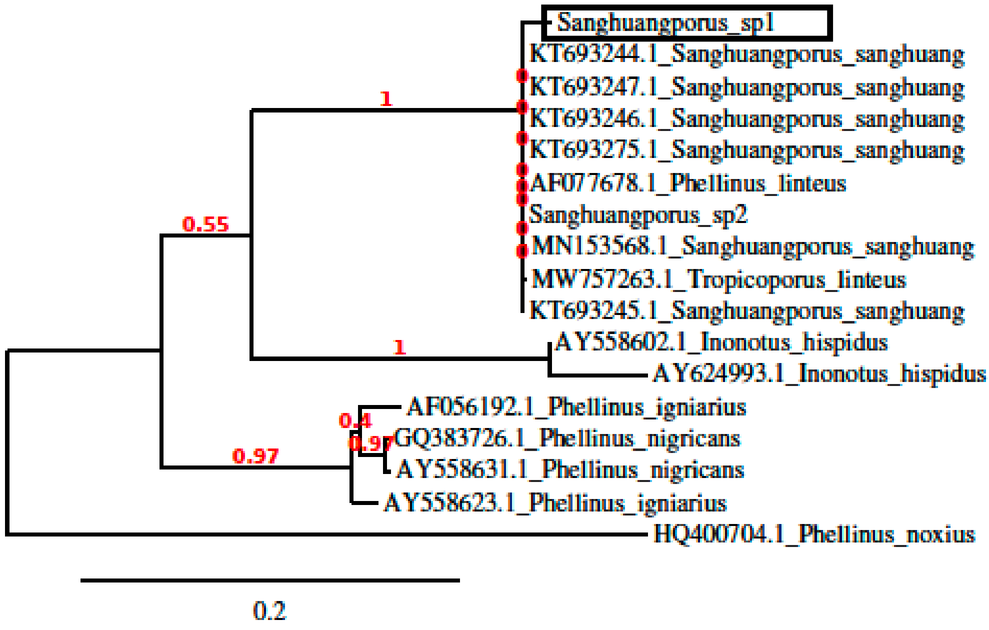

3. Polyporus squamosus (Huds.) Fr.

S1: Phylogenetic trees and morphological description of mushroom species

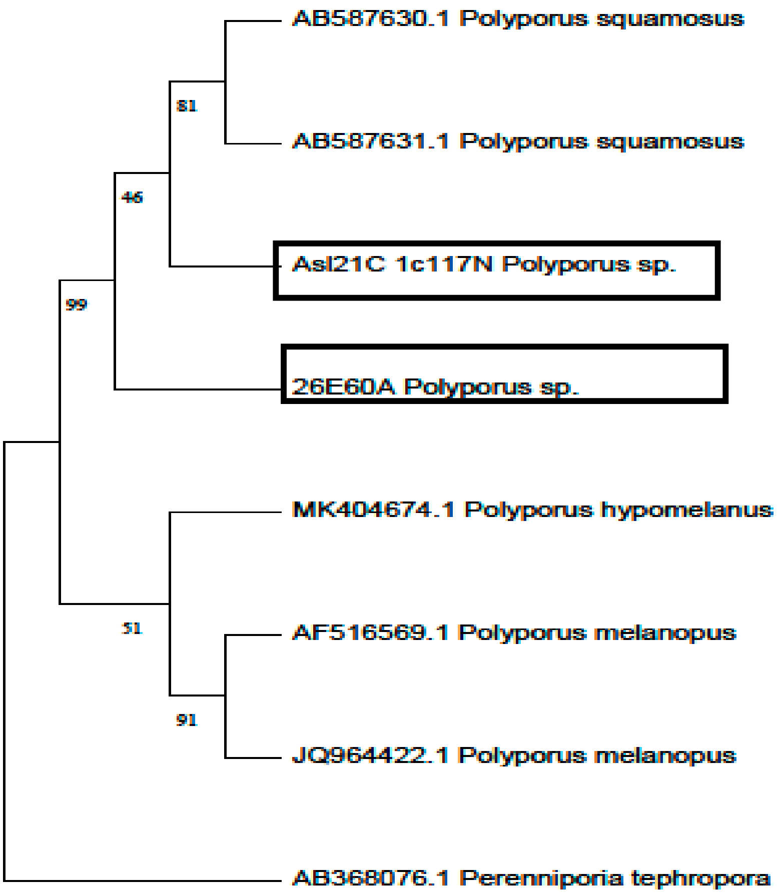

4. *Fomes fomentarius* (L.) Fr.

***GenBank Accession*=** OM663751

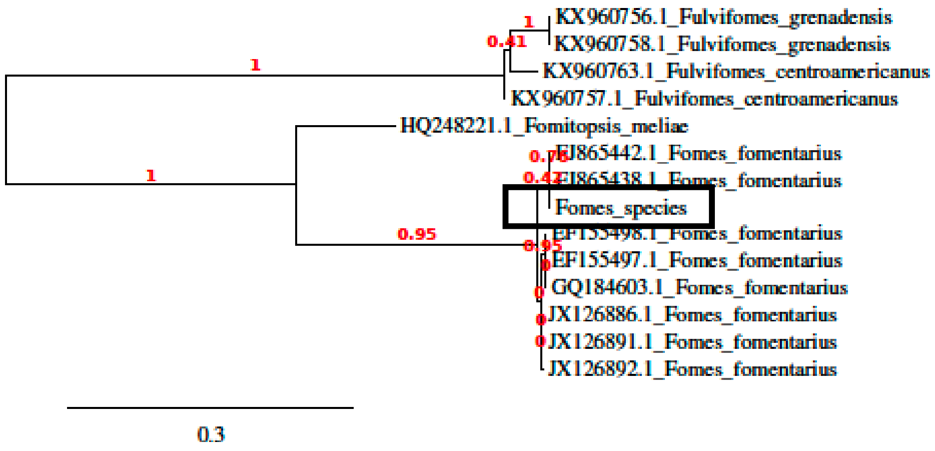

S1: Phylogenetic trees and morphological description of mushroom species

5. Fuscoporia sp

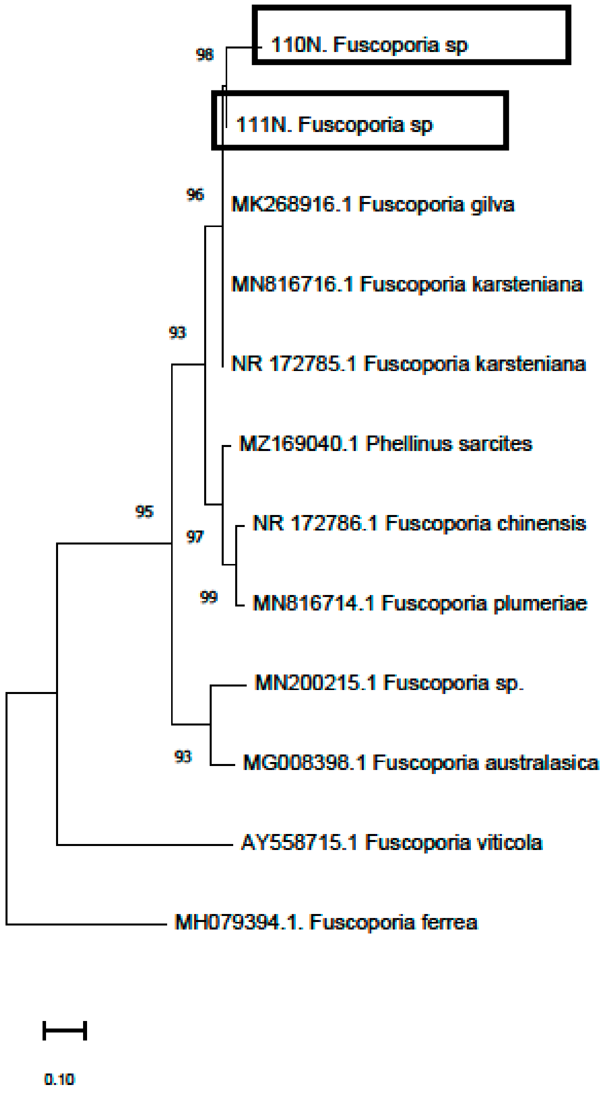

6. Laetiporus zonatus B.K. Cui & J. Song

***GenBank Accession*=** OQ300108

S1: Phylogenetic trees and morphological description of mushroom species

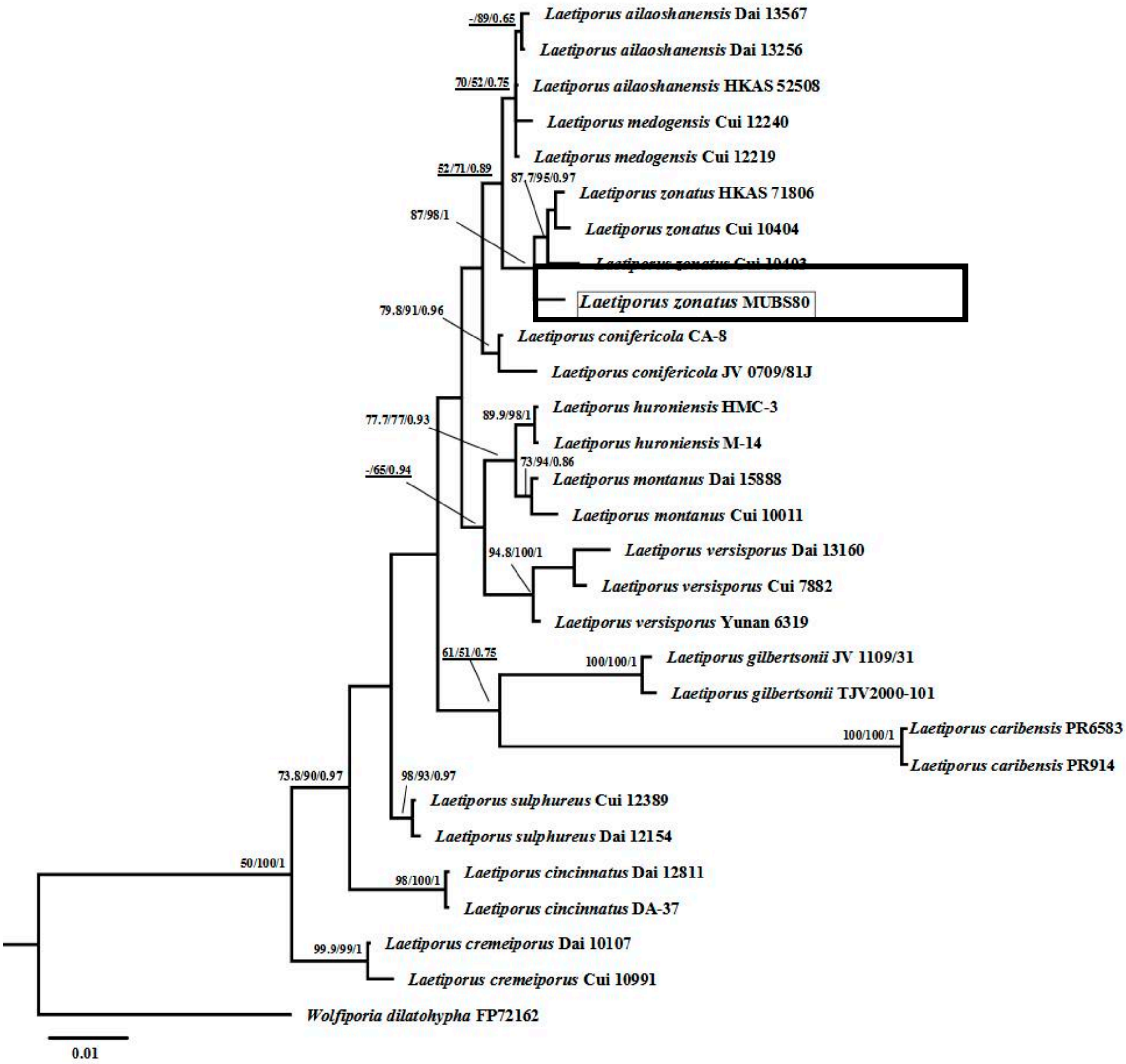

7. ***Grifola frondosa* (Dicks. Fr.) S. F. Gray**
   ***GenBank Accession*=** OQ453162
8. ***Sparassis latifolia* Y.C. Dai & Zheng Wang**
   ***GenBank Accession*=** OQ453180

S1: Phylogenetic trees and morphological description of mushroom species

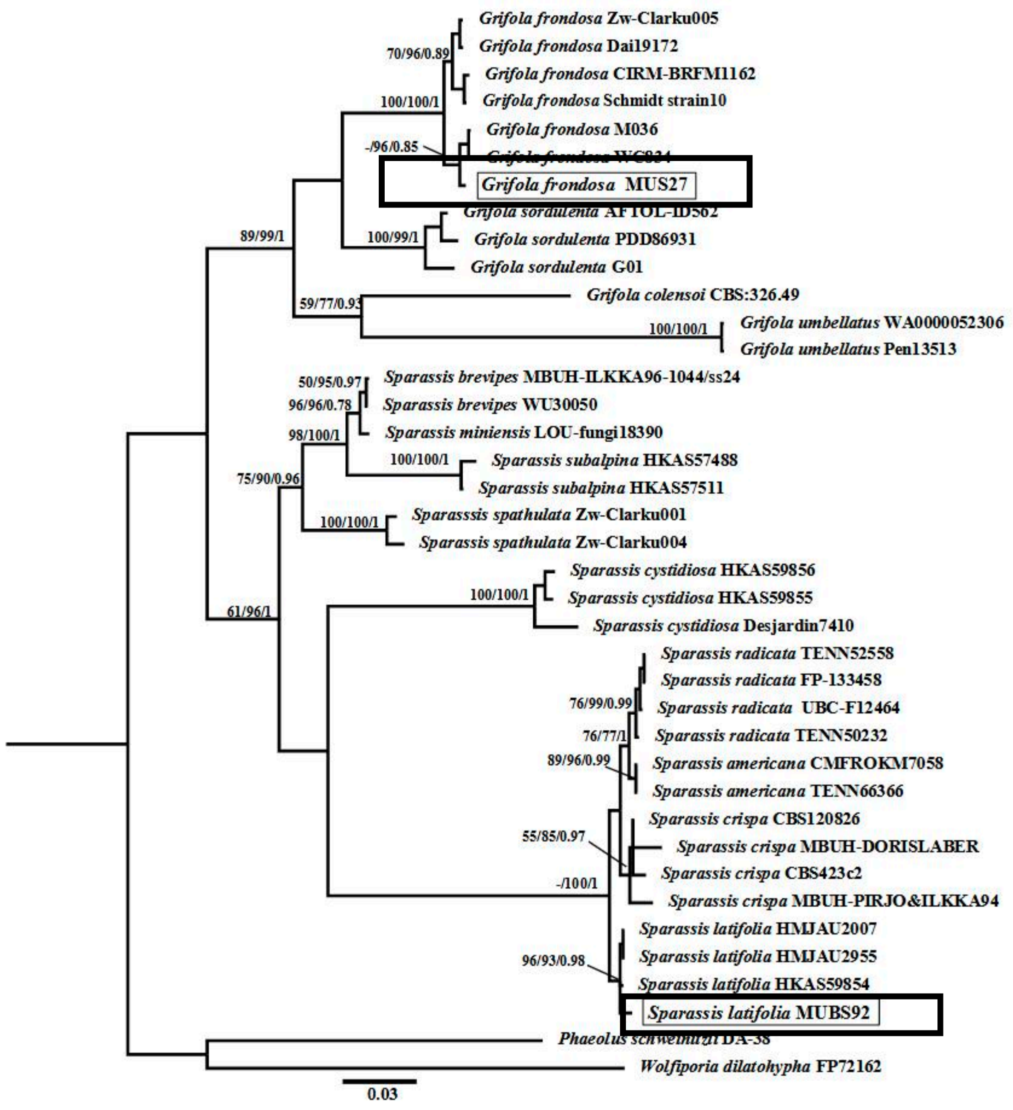

9. **Inonotus sp.**

S1: Phylogenetic trees and morphological description of mushroom species

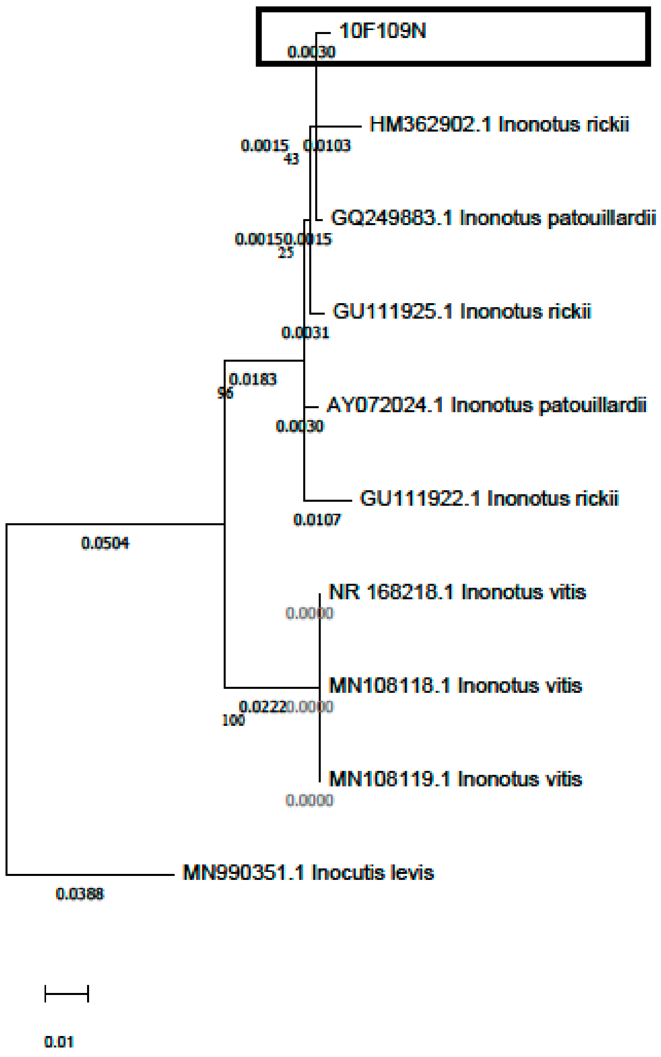

10. Ganoderma lucidum (Curtis) P. Karst.

S1: Phylogenetic trees and morphological description of mushroom species

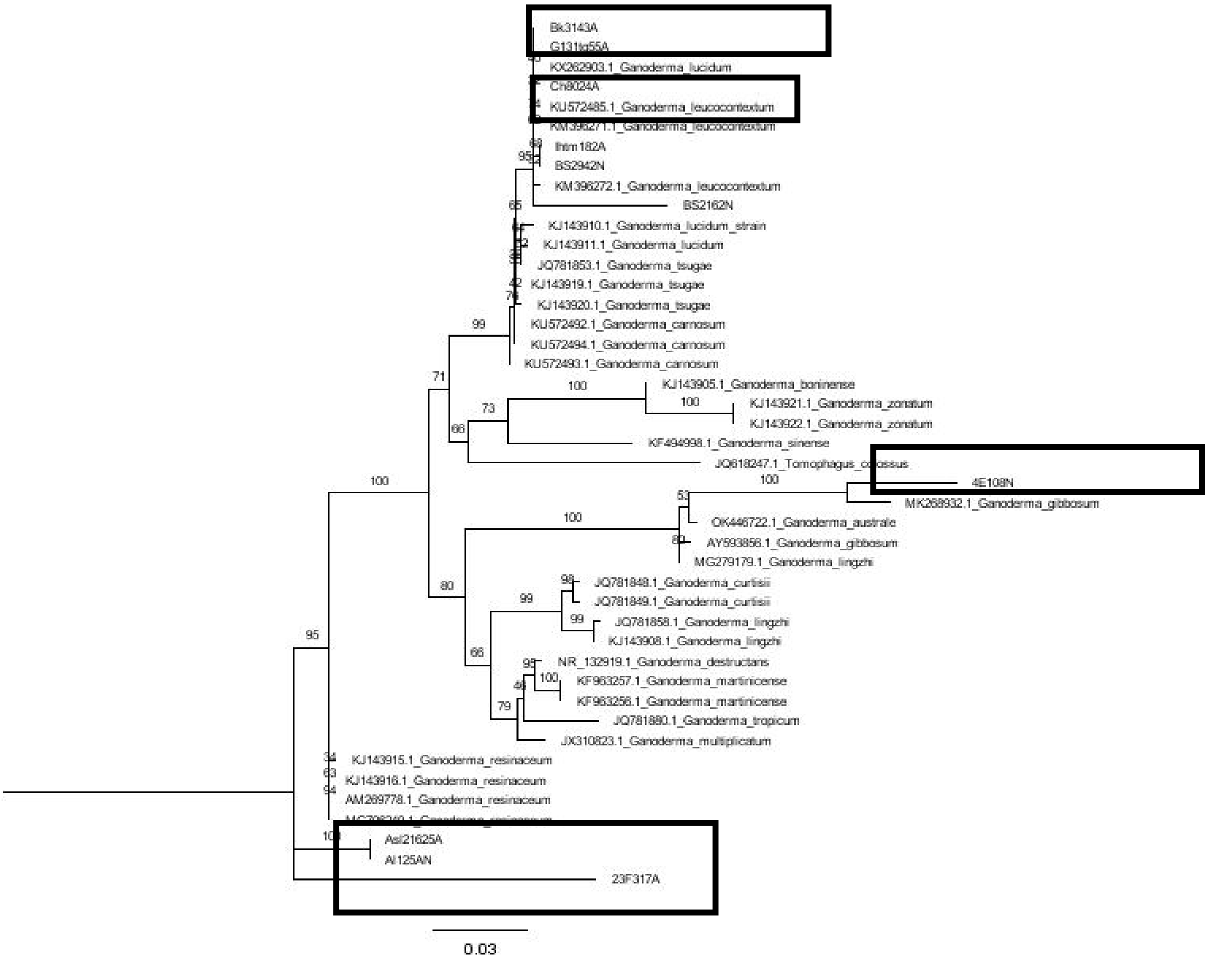

11. Phellinus nigricans (Fr.) P. Karst.

S1: Phylogenetic trees and morphological description of mushroom species

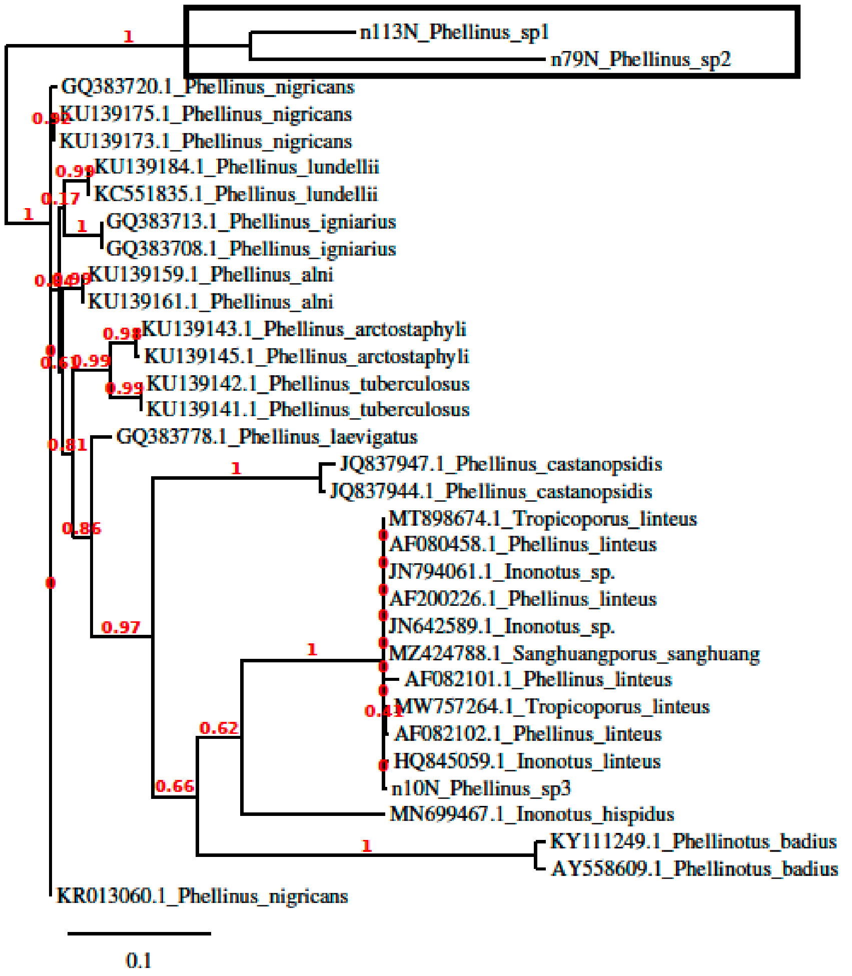

12. Pleurotus sp.

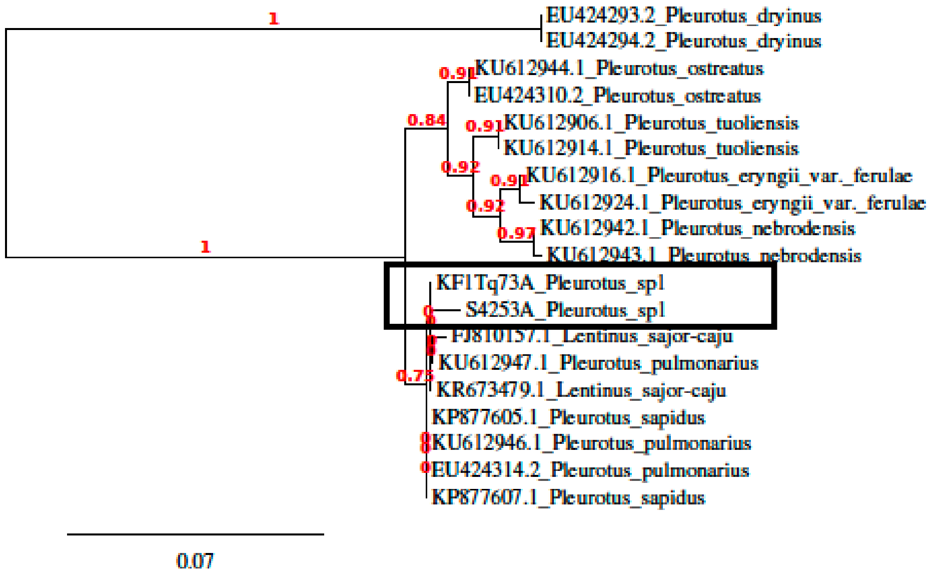

S1: Phylogenetic trees and morphological description of mushroom species

13. Auricularia sp.

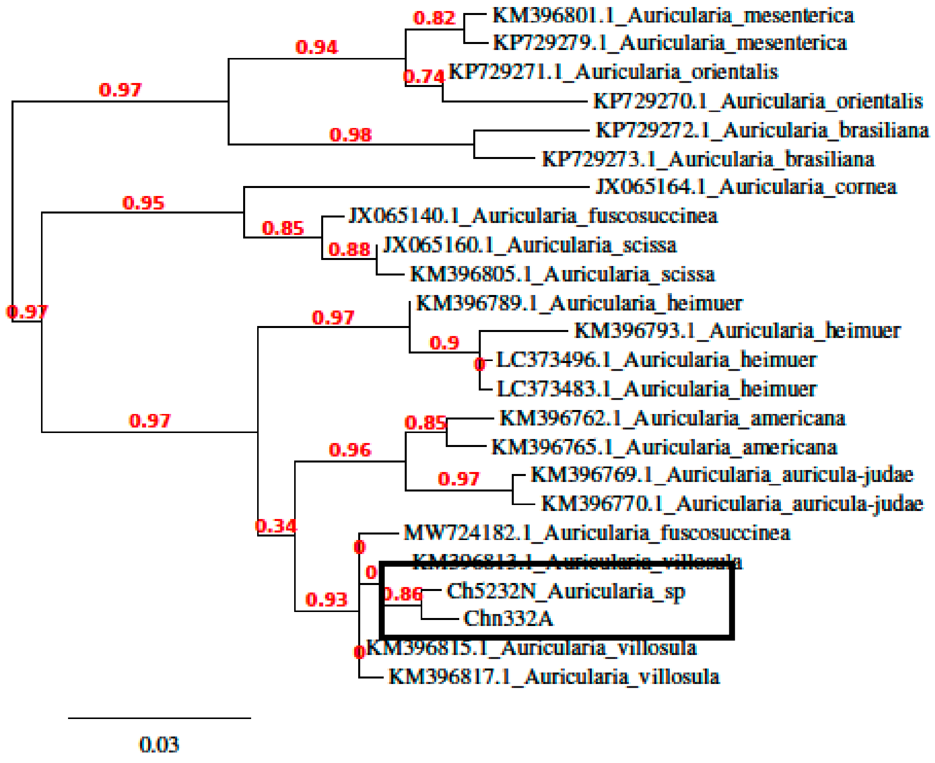

14. Hericium cirrhatum (Pers.) Nikol.

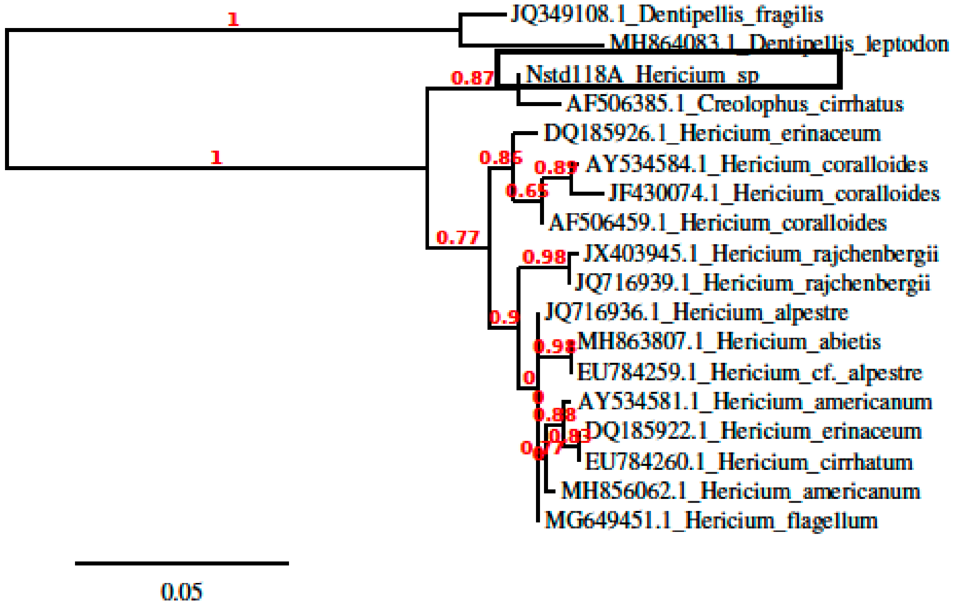

15. Gyromitra sp.

S1: Phylogenetic trees and morphological description of mushroom species

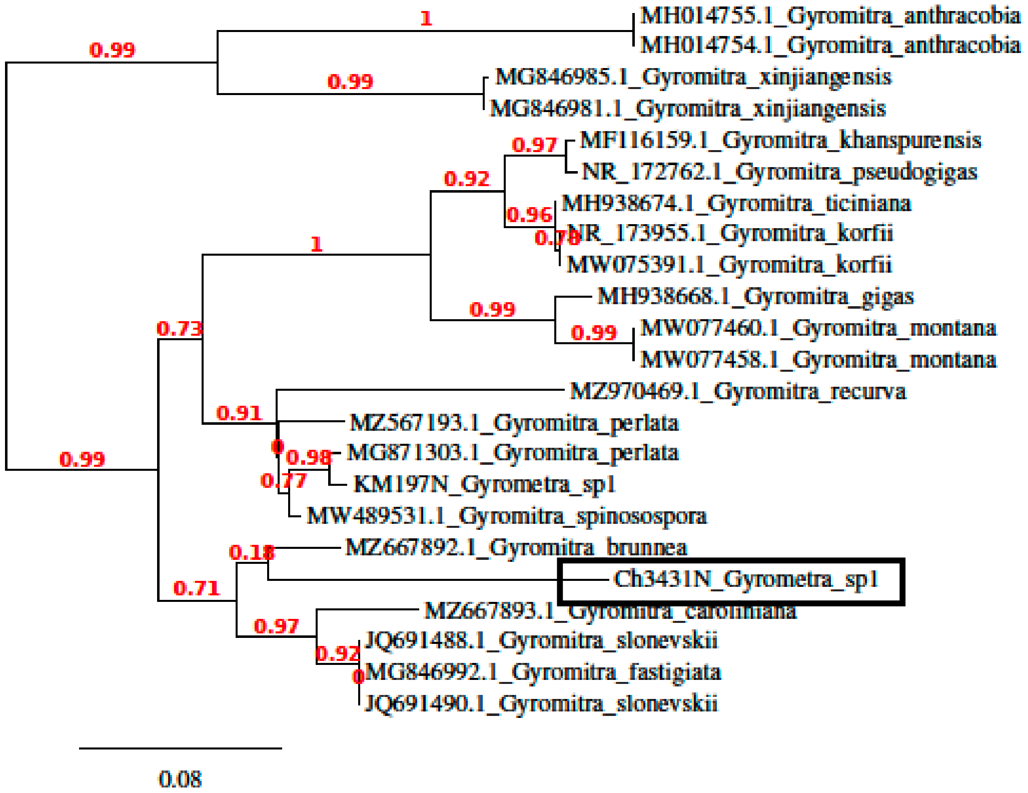

16. **Morganella pyriformis (Schaeff.) Kreisel & D. Krüger**

GenBank Accession= OM665326

17. **Phaeolus schweinitzii (Fr.) Pat.**

S1: Phylogenetic trees and morphological description of mushroom species

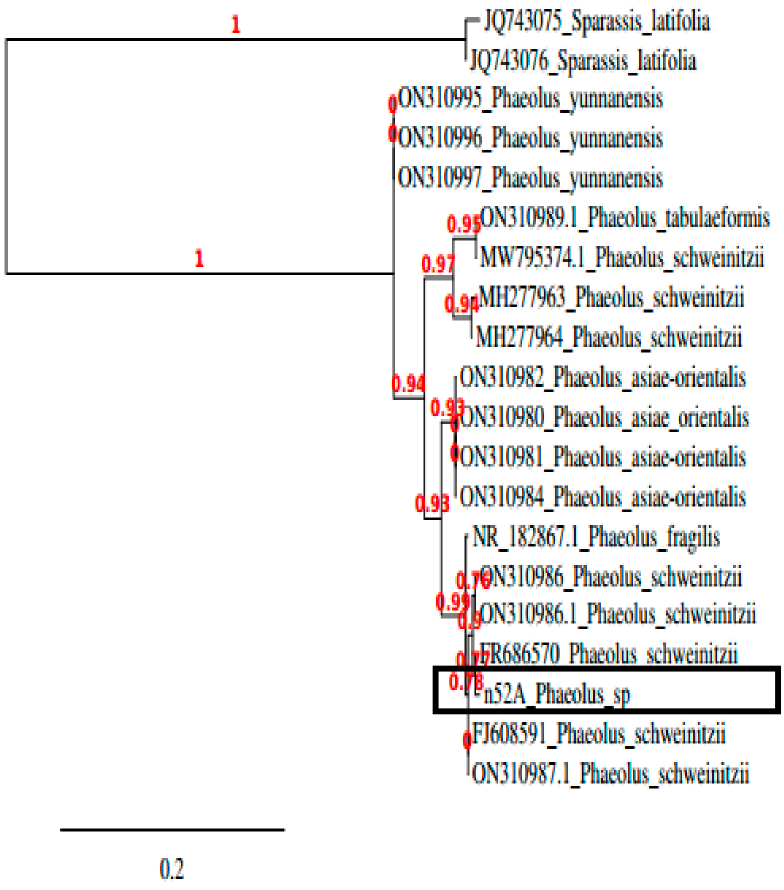

Morchella angusticepes **Peck.**

Local name: Commonly called Thora Gujai/Sukhta Gujai (Black morel)

Habitat and Growing season: Grow in mixed coniferous forest of the mountainous areas, hidden and covered by the needles. It appears very late in April extend to early June at high altitude.

Habit: Usually have solitary habit

Description: Stipe: 12-70 mm long x 6-28 mm wide, whitish or creamy pink or pale brownish colored, cylindrical, clavate or equal sometimes expanded at upper end, curved ribbed, lacunose, mealy or pubescent, become brownish hair like granules, sometimes arranged in rings, deeply grooved or lacunose base, brittle in dry condition

Hymenophore: 39-70 mm long x 20-45 mm broad, narrowly conic to ovoid or pyriform, sub-globose shaped, dark brownish to blackish , rounded, margin hanging vertically downward,

constantly looped, no sinus or leaving wide space i.e., adnate Pits: light yellowish to dark brownish colored, vertically elongated, small pits within large sized pit, protruding pit walls Ridges: more dark brown to burnt or smoky black ridges than inner of the pits, usually flattened, 0.7 to 2.5 mm thick, glabrous, 17-29 vertical ridges, little number of horizontal ridges, oblique to concave Ascospores: Spore print creamy or white, 14 -17.5 µm x 28-30.8 µm, round to elliptic shaped, appear granular due to oil contents, smooth walled Asci: cylindrical to subcylindrical, 16.8 -30 µm diameter x 204.4-308 µm length, eight spored, uniseriate arrangement Sterile elements: thin walled, stout, septate and branched or simple, 8.4 -13 µm x 114-240 µm (Fig. 4.3).

Figure 4.3. *Morchella angusticepes* and its microscopy c: Hymenial layer d: ascospores

Morchella esculenta **(L.) Pers.**

Local name: Commonly known as Ziara Gujai (Yellow morel)

Habitat and Growing season: Grow in mixed coniferous or deciduous forests, within orchards plantation such as apple, pear. It grows in April-May.

Habit: Solitary or gregarious

Description: Stipe: 42-60 mm long x 21-31 mm broad, clavate or equal, ribbed, compressed, light yellowish to white colored, mealy or slightly pubescent, stipe and head having same color, lacunose, entirely hallow, become slightly enlarged at the base

Hymenophore: 35-47 mm x 32-90 mm, subglobose to globose or ovate shaped, yellow to whitish colored, blunt conic to obtuse apex, inverted non-looped margin, no sinus, adnate at the base, possess strong fungal smell Pits: light yellow become brown with maturity, concolourous with ribs, smaller pits within a larger pit, irregular pitting, protruding walls Ridges: lighter yellow than interior of pits, irregularly disposed, 0.1 to 1mm round edges, 22 vertical ridges not distinguishable, concave horizontal ridges, highly anastomosed Ascospores: 14 -19 X 19-28 µm; rounded, granular, yellow spores deposit, hyaline singly Asci: 16.8- 22.4 x 168-300 µm, eight uniseriate spored, narrow below Sterile elements: cylindrical,14- 15.4 x 224-350 µm, septate, simple or branched at the base, thin walled (Fig. 4.6).

Figure 4.6. *Morchella esculenta* and its microscopy b: Hymenial layer c:Ascus containing ascospores d: Stipe elements

S1: Phylogenetic trees and morphological description of mushroom species

Volvariella bombycina (Schaeff.) Singer, Lilloa

The cap 50-120 mm wide and starts as an egg shape enclosed in a veil. Conical shape and becomes conico-convex to convex in shape, with a smooth surface. cap is wooly and tomentose when yount and become velvety texture and dries smooth. The cap is white to cream-colored and has a tomentose margin that exceeds the lamellae. The interior of the cap is fleshy, cream-colored, and turns slightly pinkish when cut, with a thickness of 3-4mm at the disc.

The lamellae broad (5-7mm) at the center, and light pink at first, but aging to a dark reddish-pink color. The lamellar edge can be even or serrulate, and there are lamellulae present in 1-3 tiers of diverse lengths. The stipe 40-60 x 5-12 mm, central, terete, and thickens downward with a slightly bulbous base. It is smooth and glabrous, with a fleshy, solid, wet interior that is concolorous to the exterior and unchanging. The base of the stipe is covered with a long membranous volva that is 30-40 mm high, free in the upper part, and attached at the base. The volva is white at first but becomes brownish to grayish later on. The basidiospores broadly ellipsoid to ellipsoid, rarely oval, and range from 6.8-8.3 x 4.4-5.4 µm in size. They are smooth, thick-walled (0.3-0.4 µm), and have one central guttule. The basidia are cylindrical to clavate and range from 15-20 x 5-7 µm in size. The cheilocystidia are fusiform to ventricose to more or less lageniform and range from 35-45 x 6-16 µm in size. The pleurocystidia are more or less utriform and rare, ranging from 60-71 x 10-18 µm in size. The pileipellis is a cutis, with hyphae 3-5 µm in diameter, and pileocystidia 40-50 µm long and cylindrical.

Lycoperdon perlatum Pers., Observ. mycol. (Lipsiae)

The fruiting body of this species is typically 50–70 mm wide and 40–60 mm tall, with a pear-shaped form and a distinguishable stipe, occasionally clavate. The stipe measures 20–35 mm long and 8–15 mm wide, often distorted, with a warty surface in the upper part and a granular one in the lower part. The warts, which are typically longer than 1–1.5 mm and pyramidal, appear white in young specimens and gradually develop brownish or pinkish tones (5Y 9/4) upon maturity. The warts are often fragile and senesce by maturity, leaving behind a netted peridium, which opens by

an apical pore. The gleba starts off fleshy and white, then turns powdery and olivaceous brown before drying out to a dark brown color. The basidiospores are generally globose, measuring 4.8–5.2 μm in length and 4.7–5 μm in width, with a Q value of 1–1.06 (–1.1). They are warted with warts that are blackish brown in KOH solution and have walls ≤0.5 μm thick. The peridial cells, which are sub-globose to clavate, measure 12–20 μm in length and 9–12 μm in width. They appear hyaline in KOH solution, with walls ≤0.5 μm thick.

Calocera viscosa **(Pers. ex Fr) Fr**.

The fruiting body is a coralloid, lignicolous structure that measures 40-70mm in length and 1-1.5mm in width. It is branched, with erect, forked branches that resemble antlers. The color of the branches ranges from bright yellow to orangish-yellow, with a shiny, greasy-slimy texture that is more pronounced when wet. The buried portion is whitish-yellow in color and smooth in texture. The basidiospores are ellipsoidal to more or less phaseoliforme in shape, measuring (8.7-) 9.2-11.5 (-11.8) x (4-) 4.1-5.1 (-5.2) μm in size. They are smooth-walled, with an apiculus and two central guttules, and are mostly aseptate, although some may have a single septum. The spores are hyaline in KOH solution. The basidia are bi-sterigmated and resemble a tuning fork in shape, measuring 20-25 x 3-5 μm, and are also hyaline in KOH solution. The contextual hyphae are septate, measuring 4-6 μm in diameter, with some areas inflated and others with clavate to cylindrical terminal elements.

Helvella sp.

Pileus saddle shape measures between 30 to 50 millimeters and is irregularly lobed, with an inrolled edge. The upper surface is smooth to bumpy and has a cream color, which does not change when bruised. The interior of the pileus is thin, measuring around 1 millimeter in thickness, and also cream-colored. The undersurface of the pileus is initially cream-colored and becomes light brown to brown with age. It has a fine hairy texture. The stipe, or stem, measures between 60 to 100 millimeters in length and 15 to 20 millimeters in width; attached centrally or off-centrally to the cap and starts off white to cream-colored when young. However, it develops yellowish-brown coloration as it matures. The surface of the stipe is smooth and furrowed with ribs that have blunt edges. The interior of the stipe is hollow, and the flesh is thin and pliable. The asci, which are the sacs that contain the spores, are 250 to 300 μm long and 14 to 17 μm wide. The ascospores are elliptical and measure between 17.5 to 20 μm in length and 11 to 13 μm in width. They contain a single large oil droplet in the center, and some may have a few small oil droplets as

well. The paraphyses, which are sterile cells found between the asci, are cylindrical with clavate apices and taper towards the base. They are of the same length as the asci and measure up to 3 to 6 μm in width.
